# Supplementary material for: Co-Occurrences of Forms of Child Undernutrition in India: Insights from the National Family Health Survey
Source: Nutrients. 2025 Mar 11;17(6):977. doi: 10.3390/nu17060977 (PMC11944349; doi:10.3390/nu17060977)

Supplementary Material

Table S1: Characteristics of the Study population of under-five children, NFHS-5 2019-21

	Sample Size (n)	Percentage (%)		Sample Size (n)	Percentage (%)
**Background Characteristics**			**Body Mass Index of mother**		
**Region**			Underweight	31687	18.9
North	32450	13.4	Normal	100128	55.5
Central	43499	26.6	Overweight	29187	18.0
East	34599	26.9	Missing/Currently Pregnant	13667	7.6
North-East	27153	3.8	**Diet of mother**		
West	15727	12.7	Low	60122	32.8
South	21861	16.6	Medium	79599	44.5
**Empowered-Action-Group (EAG) State**			High	35568	22.6
Non-EAG State	87770	46.4	**Mother's age at child birth**		
EAG State	87519	53.6	<19	10445	6.8
**Place of residence**			19-25	98515	58.4
Urban	35112	26.2	25-34	58728	31.5
Rural	140177	73.8	35-44	7433	3.3
**Social Group**			45+	168	0.1
Scheduled Caste (SC)	35685	23.4	**Wantedness of child**		
Scheduled Tribe (ST)	36315	10.0	No more/Later	11484	7.0
Other Backward Class (OBC)	66311	43.3	Wanted	163805	93.0
Others	36978	23.2	**Institutional delivery of child**		
**Wealth index of the household**			No	24487	11.4
Poorer	88835	46.4	Yes	150802	88.6
Middle	34222	19.8	**Birth Order (BO) of child**		
Richer	52232	33.9	1 B.O.	66350	38.7
**Toilet facility in Household**			2-3 B.O.	85911	49.4
Improved, not shared	110004	60.6	4 & more	23028	11.9
Unimproved source/Shared/Other	28607	16.6	**Birth weight of child**		
Open defecation	36678	22.8	Low	27885	16.9
**Maternal and Child Characteristics**			Normal	141548	80.1
**Education of mother**			High	3662	2.2
Illiterate	38342	21.3	Not weighed/Don't know	2194	0.9
Literate	136947	78.7	**Sex of the child**		
**Maternal height**			male	90235	51.7
Short	20023	12.0	female	85054	48.3
Normal	139728	79.4	**Child age**		
Tall	14866	8.3	6-23 months	53969	31.4
Not weighed/Missing	672	0.4	24-59 months	119844	68.6

Table S2: Estimates of adjusted odds ratios (AOR) and 95% confidence intervals (CI) from separate binary logistic regression analyses conducted to identify determinants of five outcome variables: the co-occurrences of SWUA (Stunting, Wasting, Underweight, and Anemia), SUA (Stunting, Underweight, and Anemia), SA (Stunting and Anemia), as well as "Only Anemia" and "Only Stunting," NFHS-5 (2019–21).

	SWUA		SUA		SA		Only Anemia		Only Stunting	
	AOR	95% CI	AOR	95% CI	AOR	95% CI	AOR	95% CI	AOR	95% CI
**Background Characteristics**										
**Region**										
North[®]	1.00		1.00		1.00		1.00		1.00	
Central	1.29	(1.12-1.47)***	1.03	(0.95-1.11)[ns]	1.08	(1-1.16)*	0.83	(0.79-0.88)***	1.14	(1.02-1.28)**
East	1.53	(1.33-1.75)***	0.98	(0.91-1.07)[ns]	0.80	(0.74-0.87)***	0.78	(0.74-0.83)***	0.90	(0.8-1.02)*
North-East	0.98	(0.81-1.19)[ns]	0.87	(0.78-0.97)**	0.89	(0.8-0.99)**	0.71	(0.66-0.76)***	1.38	(1.2-1.59)***
West	2.27	(1.9-2.71)***	1.39	(1.26-1.54)***	0.93	(0.84-1.03)[ns]	0.65	(0.61-0.7)***	0.80	(0.67-0.95)**
South	1.26	(1.06-1.49)***	1.08	(0.97-1.2)[ns]	0.78	(0.71-0.85)***	0.67	(0.63-0.72)***	1.11	(0.97-1.28)[ns]
**EAG State**										
Non-EAG[®]	1.00		1.00		1.00		1.00		1.00	
EAG	0.83	(0.71-0.97)**	1.02	(0.93-1.12)[ns]	0.93	(0.85-1.01)*	0.94	(0.89-1)*	1.08	(0.95-1.24)[ns]
**Place of residence**										
urban[®]	1.00		1.00		1.00		1.00		1.00	
rural	0.94	(0.82-1.07)[ns]	0.94	(0.88-1.01)*	1.02	(0.96-1.09)[ns]	1.07	(1.03-1.12)***	0.97	(0.88-1.06)[ns]
**Social Group**										
SC[®]	1.00		1.00		1.00		1.00		1.00	
ST	1.15	(1.04-1.28)***	0.94	(0.87-1)*	0.92	(0.86-0.99)**	1.04	(0.99-1.1)[ns]	0.86	(0.77-0.96)***
OBC	0.96	(0.87-1.05)[ns]	0.87	(0.83-0.92)***	0.97	(0.92-1.02)[ns]	1.00	(0.96-1.04)[ns]	1.00	(0.92-1.09)[ns]
Others	0.78	(0.69-0.88)***	0.77	(0.72-0.83)***	0.94	(0.88-1)*	1.09	(1.04-1.15)***	1.02	(0.91-1.13)[ns]
**Wealth index of the HH**										
Poorer[®]	1.00		1.00		1.00		1.00		1.00	
Middle	0.85	(0.77-0.94)***	0.81	(0.76-0.86)***	0.94	(0.88-1)*	1.20	(1.15-1.25)***	1.00	(0.91-1.09)[ns]
Richer	0.65	(0.55-0.78)***	0.60	(0.56-0.65)***	0.83	(0.77-0.89)***	1.26	(1.2-1.32)***	0.86	(0.78-0.94)***
**Toilet facility in Household**										
Improved, not shared[®]	1.00		1.00		1.00		1.00		1.00	
Unimproved source/Shared/Other	1.01	(0.91-1.12)[ns]	1.03	(0.97-1.1)[ns]	1.07	(1.01-1.14)**	1.04	(1-1.09)*	0.93	(0.84-1.02)[ns]
Open defecation	1.16	(1.06-1.26)***	1.13	(1.07-1.19)***	1.08	(1.02-1.15)***	0.96	(0.92-1)*	0.91	(0.84-1)**
**Maternal and Child Characteristics**										
**Education of mother**										
Illiterate[®]	1.00		1.00		1.00		1.00		1.00	
Literate	0.81	(0.75-0.88)***	0.76	(0.73-0.8)***	0.90	(0.86-0.96)***	1.15	(1.11-1.2)***	1.07	(0.99-1.16)[ns]
**Maternal height categorized**										
Short[®]	1.00		1.00		1.00		1.00		1.00	
Normal	0.65	(0.6-0.71)***	0.56	(0.53-0.59)***	0.83	(0.77-0.89)***	1.75	(1.66-1.85)***	0.73	(0.67-0.81)***
Tall	0.42	(0.35-0.51)***	0.31	(0.28-0.34)***	0.62	(0.55-0.69)***	2.44	(2.27-2.63)***	0.55	(0.47-0.63)***
Not weighed/Missing	2.44	(0.96-6.21)*	0.80	(0.4-1.6)[ns]	0.80	(0.29-2.22)[ns]	0.98	(0.46-2.08)[ns]	0.75	(0.19-2.9)[ns]
**Body Mass Index**										

Normal®	1.00		1.00		1.00		1.00		1.00	
Underweight	1.70	(1.54-1.88)***	1.33	(1.27-1.4)***	0.92	(0.87-0.98)***	0.79	(0.76-0.83)***	0.86	(0.79-0.94)***
Overweight	0.61	(0.53-0.7)***	0.71	(0.66-0.76)***	1.00	(0.93-1.06)^{ns}	1.18	(1.13-1.23)***	1.11	(1.01-1.22)**
Missing/Currently Pregnant	1.37	(1.21-1.54)***	1.30	(1.2-1.4)***	1.18	(1.09-1.27)***	0.88	(0.84-0.94)***	1.00	(0.9-1.12)^{ns}
**Diet of mother**										
Low	1.00		1.00		1.00		1.00		1.00	
Medium	0.99	(0.92-1.07)^{ns}	1.01	(0.96-1.06)^{ns}	0.96	(0.91-1)*	0.98	(0.94-1.01)^{ns}	1.09	(1.01-1.18)**
High	0.92	(0.82-1.02)^{ns}	0.98	(0.91-1.04)^{ns}	0.97	(0.91-1.04)^{ns}	0.96	(0.91-1)*	1.11	(1-1.23)**
**Mother's age at child birth**										
<19®	1.00		1.00		1.00		1.00		1.00	
19-25	0.80	(0.7-0.93)***	0.79	(0.73-0.87)***	0.95	(0.87-1.04)^{ns}	1.10	(1.02-1.17)***	1.06	(0.92-1.22)^{ns}
25-34	0.74	(0.63-0.86)***	0.68	(0.62-0.75)***	0.86	(0.77-0.95)***	1.16	(1.07-1.25)***	1.01	(0.87-1.17)^{ns}
35-44	0.73	(0.59-0.91)***	0.59	(0.51-0.69)***	0.81	(0.7-0.95)***	1.04	(0.93-1.16)^{ns}	1.08	(0.86-1.36)^{ns}
45+	0.91	(0.35-2.37)^{ns}	0.54	(0.27-1.08)*	0.90	(0.44-1.87)^{ns}	0.61	(0.32-1.14)^{ns}	0.38	(0.12-1.15)*
**Wantedness of child**										
No more/Later®	1.00		1.00		1.00		1.00		1.00	
Wanted	1.00	(0.88-1.13)^{ns}	0.94	(0.87-1.02)^{ns}	1.00	(0.92-1.08)^{ns}	0.97	(0.91-1.03)^{ns}	1.10	(0.97-1.26)^{ns}
**Institutional delivery**										
No®	1.00		1.00		1.00		1.00		1.00	
Yes	0.94	(0.85-1.03)^{ns}	0.90	(0.85-0.95)***	0.93	(0.87-0.99)**	1.16	(1.1-1.22)***	0.94	(0.85-1.03)^{ns}
**Birth Order**										
1 bo®	1.00		1.00		1.00		1.00		1.00	
2-3 bo	1.30	(1.2-1.4)***	1.36	(1.3-1.43)***	1.10	(1.05-1.15)***	0.88	(0.85-0.91)***	0.98	(0.91-1.05)^{ns}
4& more	1.51	(1.33-1.71)***	1.73	(1.61-1.87)***	1.28	(1.18-1.39)***	0.78	(0.73-0.82)***	1.07	(0.95-1.21)^{ns}
**Birth weight**										
Low®	1.00		1.00		1.00		1.00		1.00	
Normal	0.61	(0.56-0.66)***	0.69	(0.66-0.73)***	1.04	(0.99-1.1)^{ns}	1.39	(1.33-1.44)***	1.08	(0.98-1.18)^{ns}
High	0.61	(0.48-0.77)***	0.65	(0.57-0.75)***	0.98	(0.85-1.13)^{ns}	1.49	(1.34-1.65)***	0.92	(0.73-1.17)^{ns}
Not weighed/Don't know	0.67	(0.46-0.96)**	0.68	(0.56-0.82)***	1.09	(0.88-1.36)^{ns}	1.38	(1.17-1.63)***	1.11	(0.8-1.54)^{ns}
**Sex of the child**										
male®	1.00		1.00		1.00		1.00		1.00	
female	0.74	(0.68-0.81)***	1.02	(0.98-1.06)^{ns}	0.94	(0.9-0.98)***	1.07	(1.04-1.1)***	0.97	(0.91-1.03)^{ns}
**Child age**										
6-23 months®	1.00		1.00		1.00		1.00		1.00	
24-59 months	0.87	(0.8-0.94)***	1.27	(1.21-1.33)***	0.66	(0.63-0.69)***	0.63	(0.61-0.65)***	1.49	(1.38-1.6)***

Note: ® represents reference category; *** represents p<.01, ** represents p<.05, * represents p<0.1. ns represents not significant.

Figure S1: Bi-variate LISA and Significance Maps Showing Co-Clustering of Quadruple Burden of Child Undernutrition with lagged values of Various Covariates, NFHS-5 (2019–21)

% Children residing in rural clusters

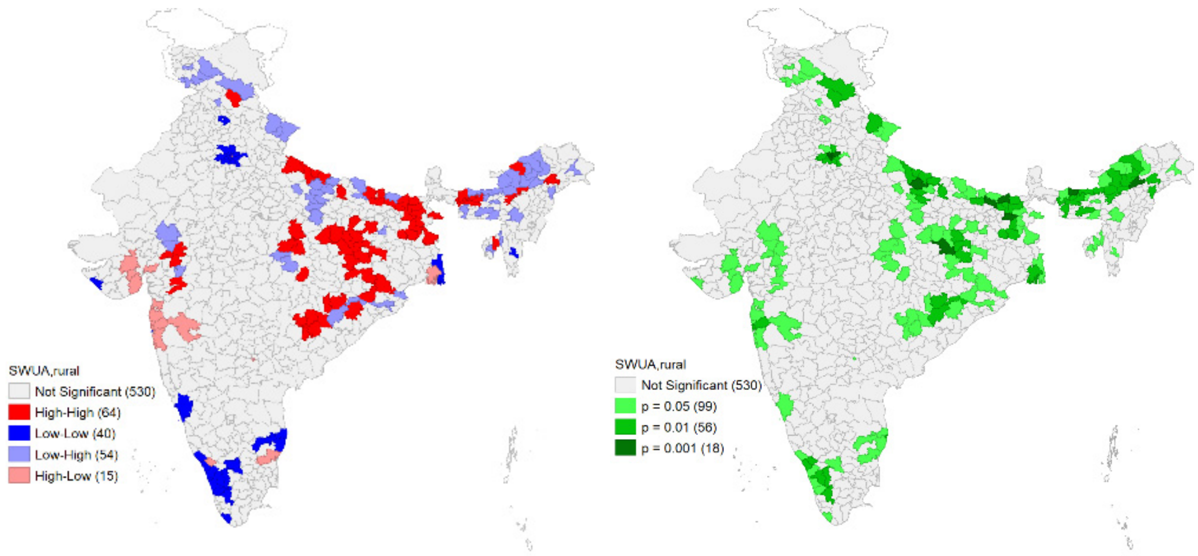

% Children from Poorer category households

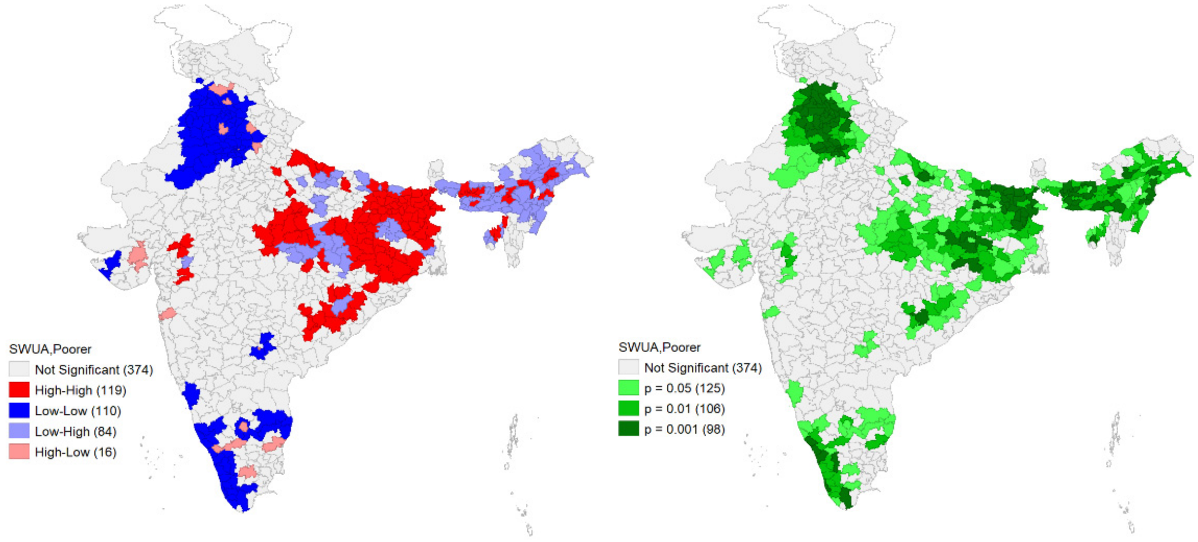

% Children belonging to household practising open defecation

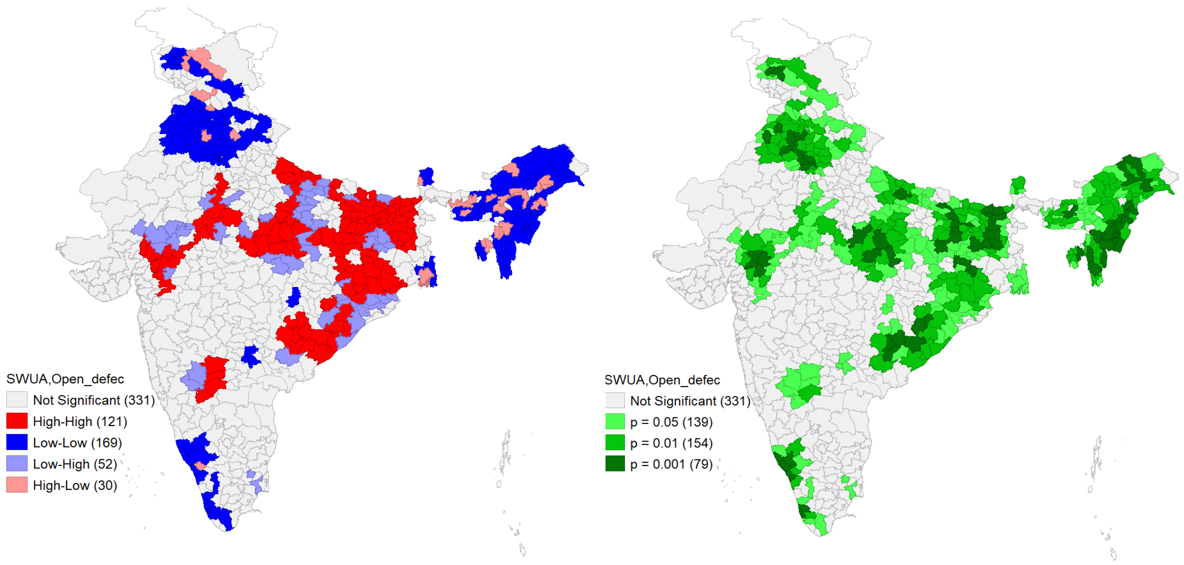

% Children from household with using clean cooking fuel

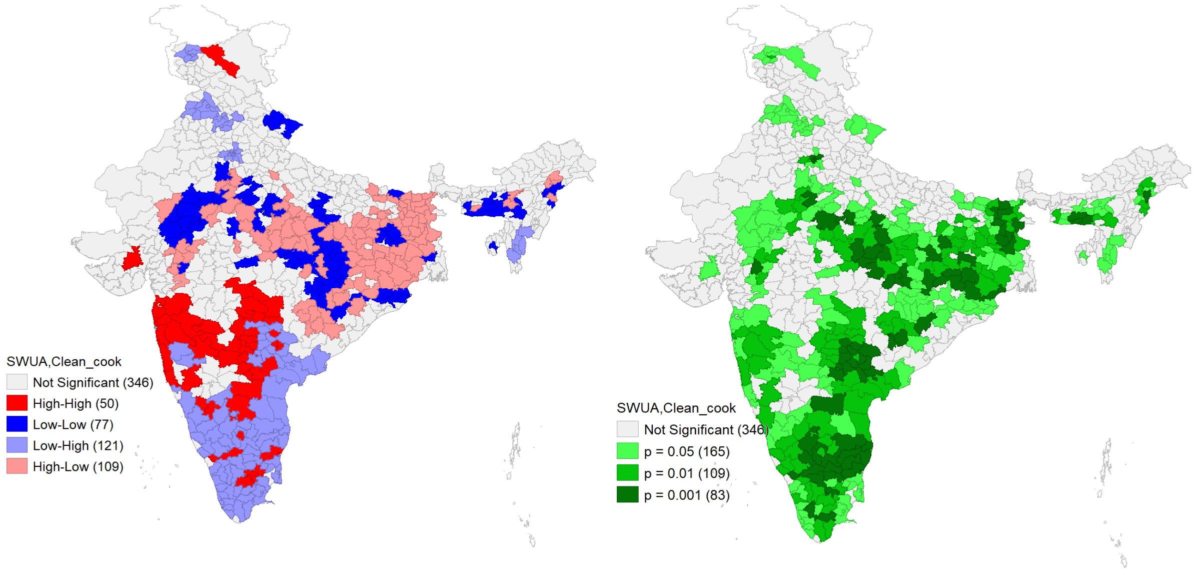

% Children whose mothers were illiterate

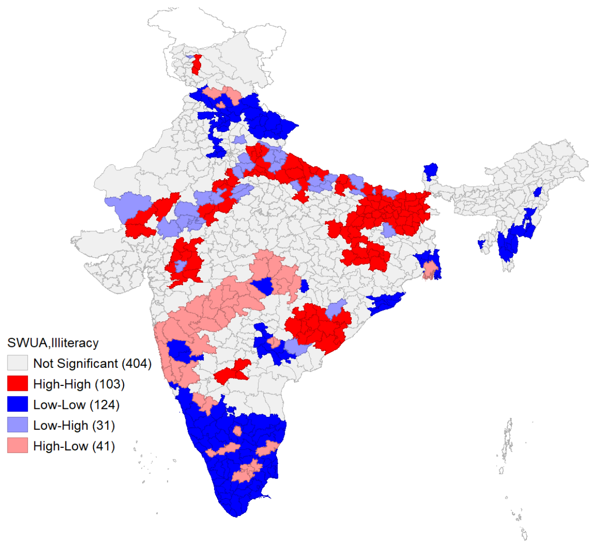

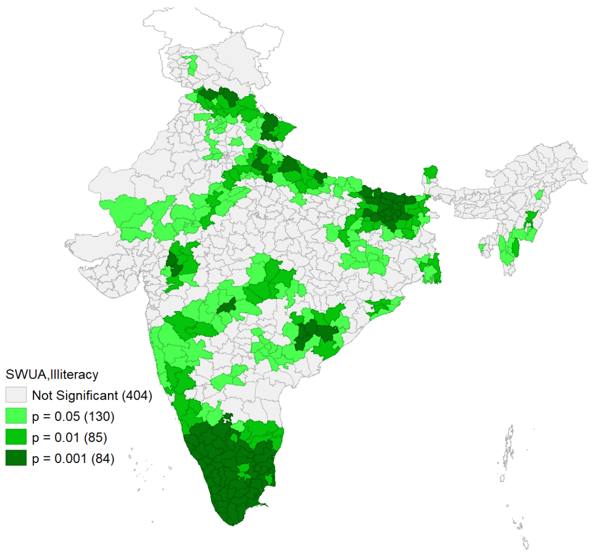

% Children whose mothers had a short height

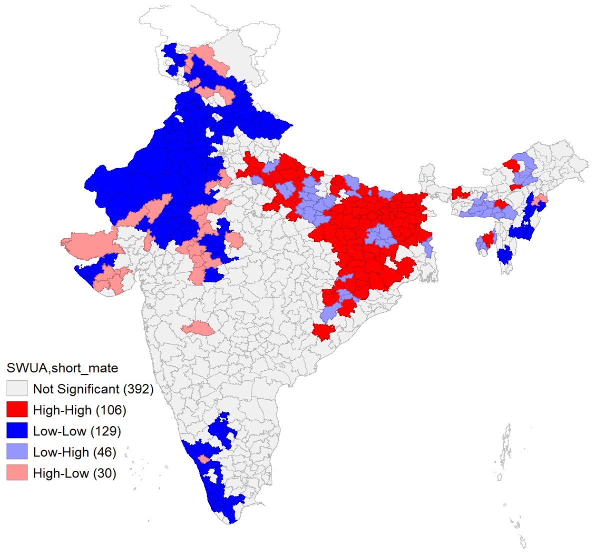

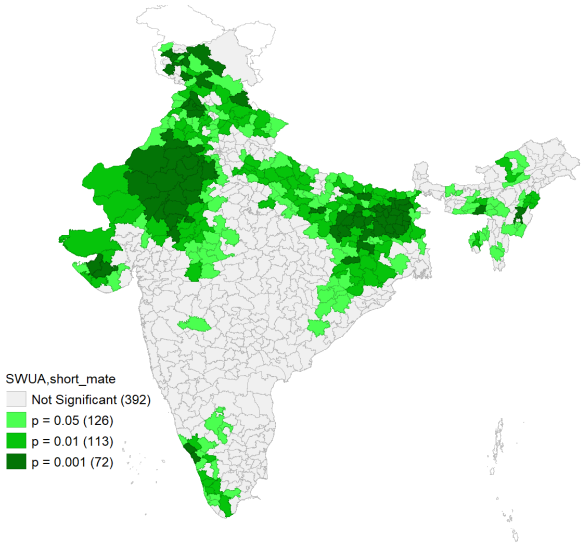

% Children with Underweight mothers

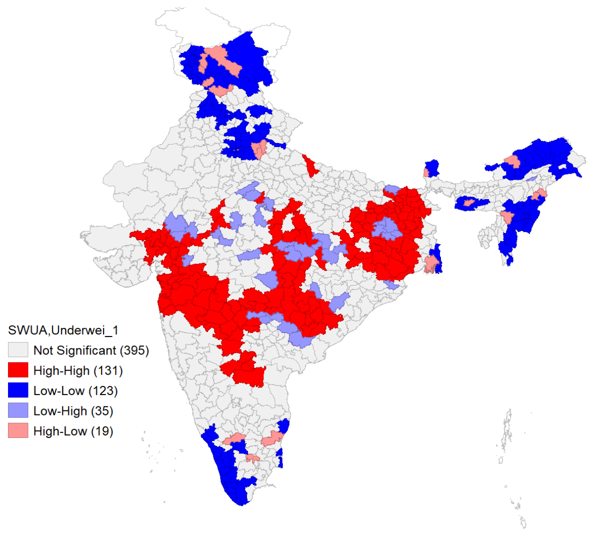

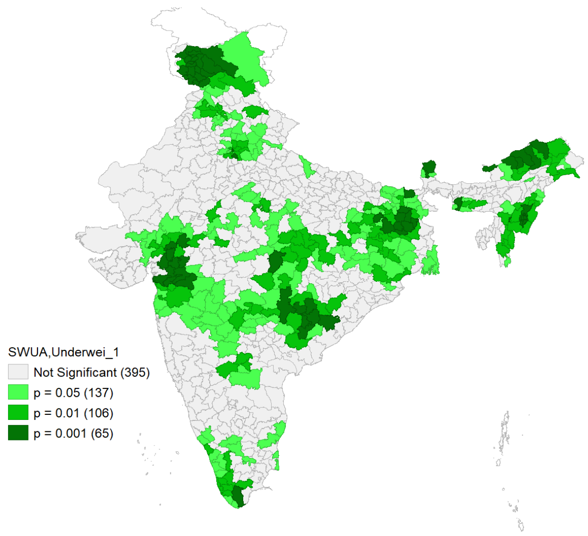

% Children whose mother's age at their birth was below 18

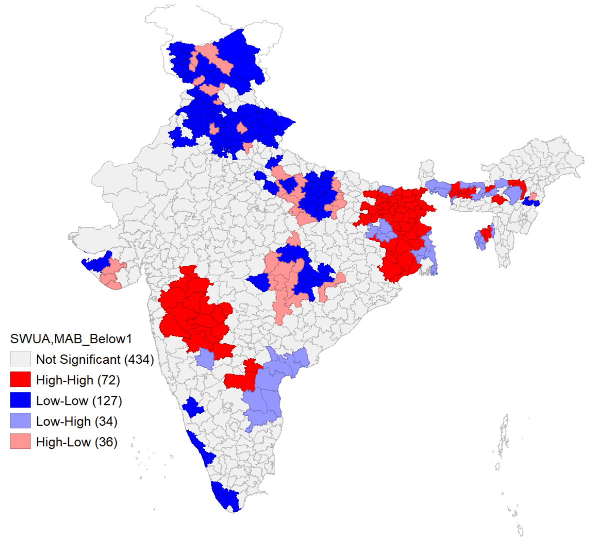

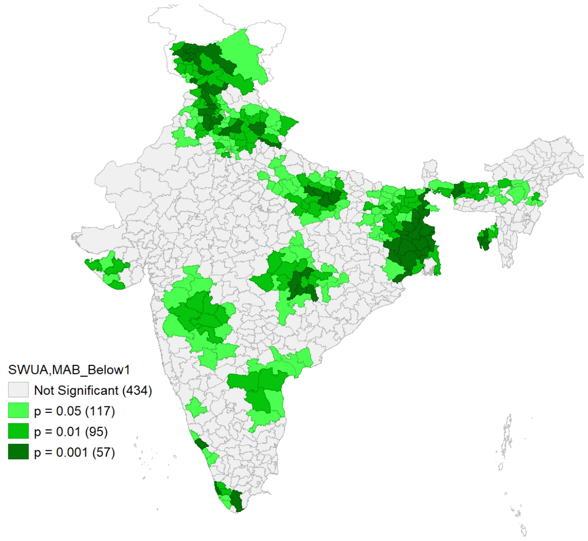

% Children with birth order 4 or more

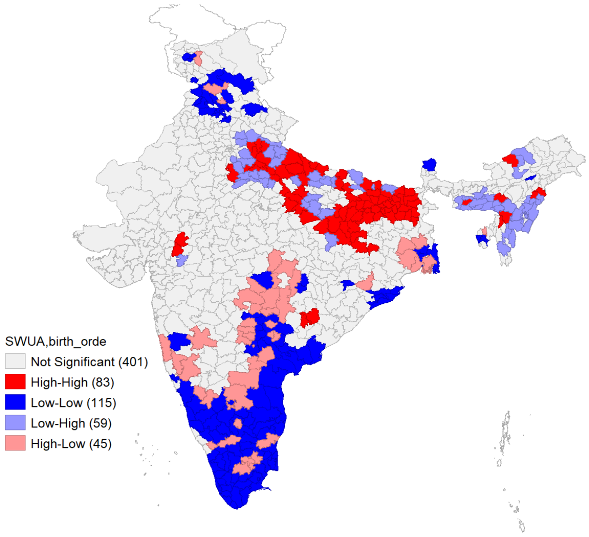

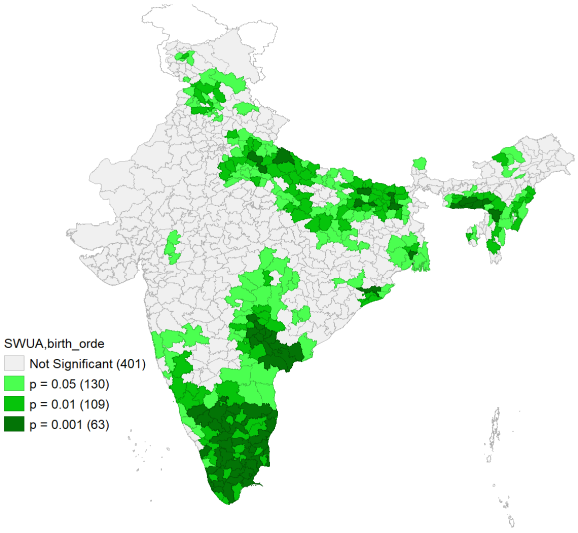

Supplement: Supplementary file 1 [file nutrients-17-00977-s001.zip › nutrients-3437124-supplementary.pdf]
